# The m.7510T>C mutation: Hearing impairment and a complex neurologic phenotype

**DOI:** 10.1002/brb3.859

**Published:** 2017-11-19

**Authors:** Laura Kytövuori, Maria Gardberg, Kari Majamaa, Mika H. Martikainen

**Affiliations:** ^1^ Research Unit of Clinical Neuroscience University of Oulu Oulu Finland; ^2^ Medical Research Center Oulu Oulu University Hospital and University of Oulu Oulu Finland; ^3^ Department of Neurology Oulu University Hospital Oulu Finland; ^4^ Department of Pathology University of Turku and Turku University Hospital Turku Finland; ^5^ Division of Clinical Neurosciences University of Turku and Turku University Hospital Turku Finland

**Keywords:** hearing impairment, mitochondrial disorder, *MT‐TS1*, neuromuscular disorder

## Abstract

**Objectives:**

Mutations in mitochondrial DNA cause a variety of clinical phenotypes ranging from a mild hearing impairment (HI) to severe encephalomyopathy. The *MT‐TS1* gene is a hotspot for mutations causing HI. The m.7510T>C mutation in *MT‐TS1* has been previously associated with non‐syndromic HI in four families from different ethnic backgrounds.

**Materials and Methods:**

We describe the clinical, genetic, and histopathological findings in a Finnish family with the heteroplasmic m.7510T>C mutation in mitochondrial DNA.

**Results:**

The family proband presented with a progressive mitochondrial disease phenotype including migraine, epilepsy, mild ataxia, and cognitive impairment in addition to HI. One young adult presented with HI only. Other family members had a mild phenotype comprising ataxia and tremor in addition to HI. Mutation heteroplasmy was 90% in the blood of maternal grandmother and ≥99% in the muscle and blood of the three other family members. Muscle histology was consistent with mitochondrial myopathy in three family members. The mitochondrial haplogroup of the family was a different branch of the haplogroup H than in the previous reports of this mutation.

**Conclusion:**

Our results suggest that, in addition to sensorineural HI, the m.7510T>C mutation is associated with a spectrum of mitochondrial disease clinical features including migraine, epilepsy, cognitive impairment, ataxia, and tremor, and with evidence of mitochondrial myopathy.

## 1 INTRODUCTION

Pathogenic mitochondrial DNA (mtDNA) point mutations are an established genetic cause of non‐syndromic hearing impairment (HI) (Prezant et al., 1993). The m.1555A>G mutation in *MT‐RNR1* gene is considered the most common mtDNA mutation associated with HI. The minimum prevalence of the m.1555A>G mutation has been estimated to be 4.7 per 100,000 in Northern Finland (Lehtonen, Uimonen, Hassinen, & Majamaa, 2000). Furthermore, HI is a prevalent feature among patients with various multisystemic mitochondrial disease phenotypes. The m.3243A>G mutation in *MT‐TL1*, initially described in association with the MELAS (Mitochondrial Encephalomyopathy, Lactic Acidosis, and Stroke‐like episodes) syndrome (Goto, Nonaka, & Horai, 1990), in fact more commonly results in a milder phenotype consisting of sensorineural HI and diabetes mellitus (Nesbitt et al., 2013). Indeed, 7.4% of the patients with symmetric sensorineural HI have been found to carry m.3243A>G in Finland (Majamaa et al., 1998), while the mutation was found to be less frequent among British patients with postlingual HI (Jacobs et al., 2005). Instead, mutations in *MT‐TS1* are more important cause of HI as they have been found with a frequency of 2.6% in Italy and 3.75% in the UK among patients with this condition (Jacobs et al., 2005).

In addition to the m.3243A>G mutation, syndromic features have been described in patients harboring mutations that are primarily associated with non‐syndromic HI. The m.1555A>G mutation has been reported to result in a multisystemic disorder as well as in cardiomyopathy without HI (Santorelli et al., 1999; Bannwarth et al., 2013) Mutations in the consecutive acceptor stem positions 7510, 7511, and 7512 of the *MT‐TS1* gene have been linked with a mitochondrial disease. Mutations in positions 7510 and 7511 have been reported to cause non‐syndromic HI (Sue et al., 1999; Hutchin et al., 2000), whereas the m.7512T>C mutation has been reported in patients with a MERRF (Myoclonic Epilepsy with Ragged‐Red Fibers)/MELAS overlap syndrome (Nakamura et al., 1995) and in patients with a syndromic phenotype with epilepsy, ataxia, cognitive impairment, and HI (Jaksch et al., 1998).

We describe a Finnish family harboring the m.7510T>C mutation with high level of heteroplasmy that resulted in a neurologic mitochondrial disease phenotype, contrasting the non‐syndromic HI previously reported in association with this mutation. Three out of four investigated family members presented with a variable neurologic phenotype including features of migraine, ataxia, tremor, epilepsy, and cognitive impairment, whereas only one young individual had non‐syndromic HI. Moreover, in the three patients with neurologic involvement, muscle histopathological investigations revealed evidence of mitochondrial myopathy.

## 2 MATERIALS AND METHODS

### 2.1 Case reports

**Figure 1:**
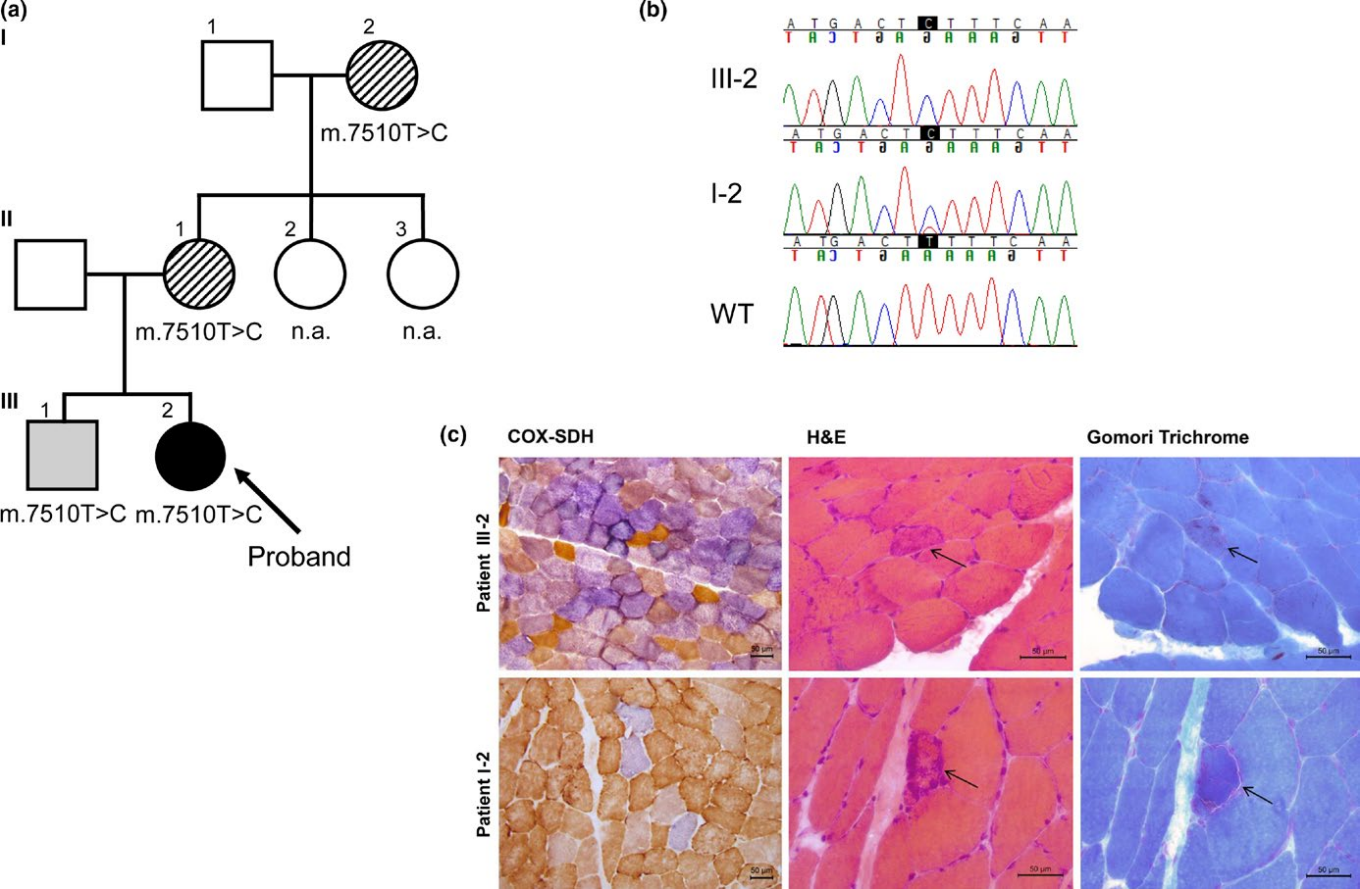
Mutation in *MT‐TS1* in a family with mitochondrial disorder. (a) Family pedigree. Solid symbol, proband with a complex neurologic phenotype; lined symbol, family member with neurologic symptoms in addition to HI; grey symbol, pure HI; open symbol, data not available (n.a.). (b) Sequence chromatograms showing variable heteroplasmy. WT, wild type. (c) Histochemical stainings of samples from *m. vastus lateralis*. COX‐SDH, cytochrome c oxidase and succinate dehydrogenase; H&E, hematoxylin and eosin. Arrow indicates a ragged‐red fiber

**Figure 2:**
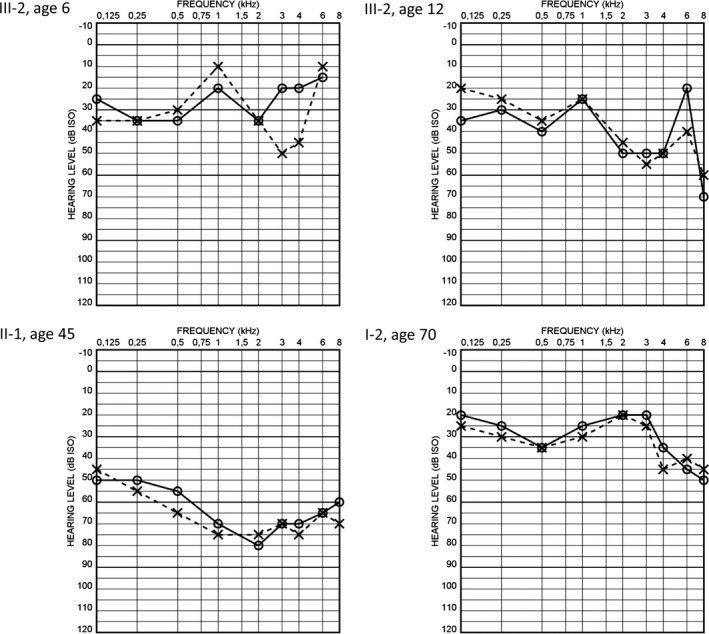
Audiograms of the family members harboring the m.7510T>C mutation. Audiograms represent pure tone hearing thresholds of right (O) and left (X) ear at all measured frequencies (kHz). III‐2, proband; II‐1, mother of the proband; I‐2, grandmother of the proband

The proband (Figure 1: III‐2) was diagnosed with sensorineural HI at the age of 6 years. Pure tone average (PTA) of the better ear hearing level at 0.5, 1, 2, and 4 kHz (BEHL 0.5–4 kHz) was 28 dB (Figure 2). A mild delay of motor skills was also noted. At the age of 9 years, she developed light‐sensitive generalized epilepsy and started having absence seizures at increasing frequency. Electroencephalography at age 9 years was abnormal and consistent with generalized epilepsy. Clinical examination at age 10 years revealed motor clumsiness and deep tendon reflexes were diminished. She started using a hearing aid at the age of 11 years, when BEHL 0.5–4 kHz was 34 dB. Speech discrimination score was 92% in her right ear and 88% on the left with the level of 60 dB. At the age of 12 years, a neuropsychological examination performed because of the observed difficulties in writing and in speech recognition revealed cognitive delay. In clinical examination at the age of 17 years, the patient had short stature, mildly ataxic gait, and hand clumsiness. Deep tendon reflexes were diminished and plantar responses were in flexion. The cognitive impairment had progressed to the level of intellectual disability. Her epilepsy was initially treated with sodium valproate which was replaced with levetiracetam after a suspicion of mitochondrial disorder was raised. Brain magnetic resonance imaging was normal. Blood lactate was 1.9 mmol/L (laboratory reference: 0.6–2.2 mmol/L) and pyruvate was 107 μmol/l (40–70 μmol/L). A next generation sequencing panel testing of 38 genes associated with epilepsy (Center of Genomics and Transcriptomics, Tübingen, Germany), as well as analysis for the common m.3243A>G, m.8344A>G, and m.8993T>G/C mutations in mtDNA and analysis of the *POLG* gene revealed no pathogenic changes.

The mother (II‐1) of the proband was diagnosed with sensorineural HI during elementary school. She started using a hearing aid in her mid‐twenties. The HI was reported to be slowly progressive. Her BEHL 0.5–4 kHz was 69 dB in the latest audiometry at the age of 45 years. Earlier audiograms were not available. Neurologic clinical examination at age 46 years revealed mild ataxia that resulted in problems with balance.

The maternal grandmother (I‐2) of the proband was investigated at the age of 70 years and was clinically affected with postural hand tremor, restless legs syndrome, and ataxia that resulted in balance difficulties in addition to sensorineural HI. PTA of 0.5–4 kHz was 29 dB in her better ear. Speech discrimination score was 100% in her right ear and 96% in her left ear with the level of 50 dB. The audiogram configuration was atypical for age‐related HI and suggestive of a hereditary type HI.

The elder brother (III‐1) of the proband was diagnosed with sensorineural HI at the age of 20 years. Audiograms were not available for review. He was otherwise clinically healthy, and neurologic examination at the age of 21 years was normal.

Family history revealed that the mother of the proband had two sisters, one of whom was currently healthy and the other was affected with restless legs syndrome. Unfortunately, these maternal aunts (II‐2, II‐3) were not available for detailed investigations.

### 2.2 Molecular methods

As the clinical features as well as the maternal inheritance pattern were suggestive of a mitochondrial disorder, the entire mtDNA was sequenced. The proportion of the mutant genome in all available tissues of the family members was determined by cloning (Martikainen, Kytövuori, & Majamaa, 2013). One hundred colonies per sample were screened for the m.7510T>C mutation using *HinfI* restriction endonuclease (Thermo Fisher Scientific, Waltham, MA, U.S.A.). The mtDNA haplogroup of the family, as well as those of the previously reported cases, was determined, and phylogenetic tree of the sequences was drawn using Haplogrep2 software (Kloss‐Brandstatter et al., 2011; van Oven & Kayser, 2009).

### 2.3 Muscle histology

Lower limb muscles were biopsied and fresh‐frozen. Cryostat sections (10 μm) were stained with routine histochemical techniques (Dubowitz, Sewry, & Oldfors, 2006; Raheem, Huovinen, Suominen, Haapasalo, & Udd, 2010). The stainings included hematoxylin and eosin, modified Gomori trichrome, reduced nicotinamide adenine dinucleotide‐tetrazolium reductase (NADH‐TR), combined cytochrome c oxidase and succinate dehydrogenase (COX‐SDH), Congo red and immunohistochemical myosin heavy chain double staining for separation of fiber types.

### 2.4 Ethical statement

High standard of ethics according to the WMA Declaration of Helsinki was applied in all investigations and clinical work described in this manuscript. The research project was approved by the Ethics Committee of Turku University Hospital. All subjects gave their written informed consent.

## 3 RESULTS

The four family members harbored the m.7510T>C transition in *MT‐TS1*. Mutation heteroplasmy was ≥99% in the blood and muscle of the proband. The heteroplasmy level was similar in the blood of the brother as well as in the blood and buccal mucosa of the mother, while the proportion of the mutation was 90% in the blood of the maternal grandmother.

The proband belonged to mtDNA haplogroup H13a1a1d1 (Appendix S1). The previously reported Spanish family (del Castillo et al., 2002) belonged to haplogroup H1ar and the North American Caucasian family (Labay et al., 2008) to haplogroup H24a1. The population sequence (Soares et al., 2011) (ID: HQ873560.1) in GenBank represents haplogroup B4a1a3a.

Histologic examination of muscle of the proband at the age of 17 years revealed clearly pathologic findings. The majority of muscle fibers were COX‐negative (and SDH positive) in the COX‐SDH staining. NADH‐TR staining was intense at the periphery of several fibers, suggesting mitochondrial proliferation (not shown), but only a single RRF was found (Figure 1c). Muscle of patient II‐1 at the age of 46 years had subtle histologic alterations. An increased number of COX‐negative fibers was found (4%), but no RRFs were present. NADH‐TR staining was normal. In patient I‐2, a clearly increased number of COX‐negative fibers (10%) as well as several RRFs was found at the age of 70 years (Figure 1c). NADH‐TR staining was indicative of mitochondrial proliferation. Slight small group atrophy and fiber type grouping was also present, suggesting mild neurogenic change.

## 4 DISCUSSION

We describe a Finnish family with the m.7510T>C mutation and the associated variable phenotype in four affected family members. The m.7510T>C mutation has previously been reported in four families (Hutchin et al., 2000; del Castillo et al., 2002; Labay et al., 2008; Komlósi et al., 2013). In two families, the phenotype was an isolated HI (del Castillo et al., 2002; Labay et al., 2008) while in the other two families additional neurologic symptoms were described (Hutchin et al., 2000; Komlósi et al., 2013). In the Hungarian family, the proband was reported to have coordination problems and delayed fine motor skills in his first clinical examination at the age of 4 years (Komlósi et al., 2013) Besides clumsiness, possibly indicative of ataxia, the neurologic examination was unremarkable. Intellectual disability was reported in the maternal aunt of the proband in a UK family, but interpreted as being due to birth asphyxia (Hutchin et al., 2000).

Based on the reported cases, both the age of onset and severity of HI related to the m.7510T>C mutation are variable. In the Hungarian family, the only person unaffected by HI was only 3 years old (Komlósi et al., 2013) In the Spanish family, three unaffected persons were 11–16 years of age, and the fourth unaffected family member was already 31 years old at the time of investigations (del Castillo et al., 2002). When considering the wide range in the age of onset related to this mutation, the clinical phenotype thus far associated with this mutation might still be incomplete. The level of heteroplasmy does not seem to explain the phenotypic variability as the mutation has been described in homoplasmic state in three of the four previously reported families (del Castillo et al., 2002; Labay et al., 2008; Komlósi et al., 2013). In our study, patient I‐2 was clearly heteroplasmic and her HI was mild. However, she had other symptoms in addition to her HI. Phenotypic variability is a common finding in patients harboring a mutation in *MT‐TS1* regardless of the mutation load (Chapiro et al., 2002) Frequently, the genotype phenotype correlation exists between heteroplasmy of tRNA mutations and clinical presentation, but in many patients the correlation cannot be recognized (Morgan‐Hughes et al., 1995; Virgilio et al., 2009).

Haplogroup analyses of the patients harboring m.7510T>C have been previously reported to support the pathogenicity of the mutation (Labay et al., 2008) Our patient belonged to haplogroup H13a1a1d1, a subhaplogroup H different from those in previously reported patients and thus further supporting the pathogenicity of this mutation (Appendix S1). Moreover, the mutation has been reported in subhaplogroup B4 (Soares et al., 2011).

Interestingly, the *MT‐TS1* gene encoding tRNASer^UCN^ seems to be a hotspot for pathogenic mutations. MITOMAP database (http://www.mitomap.org/MITOMAP) includes 15 disease‐associated mutations reported at least once. The acceptor stems of the different mitochondrial tRNAs are rarely mutated. It is quite exceptional that three sequential base pairs of the acceptor stem are associated with disease phenotype, as is the case for the mutations m.7510T>C, m.7511T>C, and m.7512T>C in the tRNASer^UCN^. The three mutations have been shown to reduce tRNase Z^L^ processing efficiency and create structural alterations of the acceptor stem (Yan, Zareen, & Levinger, 2006). The m.7510T>C mutation was estimated to be the most deleterious among these three mutations.

A complex neurologic mitochondrial disease phenotype has not been reported previously in association with m.7510T>C. There is a previous report of this mutation detected in a cohort of patients with ataxia, but no clinical details were reported (Bargiela et al., 2015). In the family reported here, muscle histology was consistent with a mitochondrial myopathy. Abnormal mitochondrial morphology as well as decreased COX activity have previously been reported in few patients with m.1555A>G mutation indicating a wider spectrum of pathologies resulting from HI‐associated mutations (Yamasoba et al., 2002; Kouzaki, Suzuki, & Shimizu, 2007. The phenotype of the proband consisted of HI, migraine, ataxia, short stature, epilepsy, and cognitive impairment, whereas the other family members presented with a variable phenotype ranging from non‐syndromic HI to complex, syndromic presentations with mild ataxia, tremor, restless legs syndrome, and HI. Ataxia is a common clinical manifestation in patients with mitochondrial disorder, (Lax et al., 2012) while various movement disorder presentations including tremor and restless legs syndrome are increasingly recognized in the context of mitochondrial disease (Martikainen et al., 2016).

In conclusion, our results suggest that the m.7510T>C mutation is associated with a variable mitochondrial disease phenotype ranging from non‐syndromic HI to more severe but variable neurologic presentations with clinical features including ataxia, tremor, epilepsy, and cognitive impairment. The present study also highlights the value of meticulous clinical investigations and family tracing when establishing a diagnosis of a mitochondrial disorder and the associated phenotypic range.

## CONFLICT OF INTEREST

The authors declare no conflicts of interest.
