# Supplementary material for: The m.7510T>C mutation: Hearing impairment and a complex neurologic phenotype
Source: Brain Behav. 2017 Nov 19;7(12):e00859. doi: 10.1002/brb3.859 (PMC5745241; doi:10.1002/brb3.859)
Supplement: Supplementary file 1 [file BRB3-7-e00859-s001.pdf]

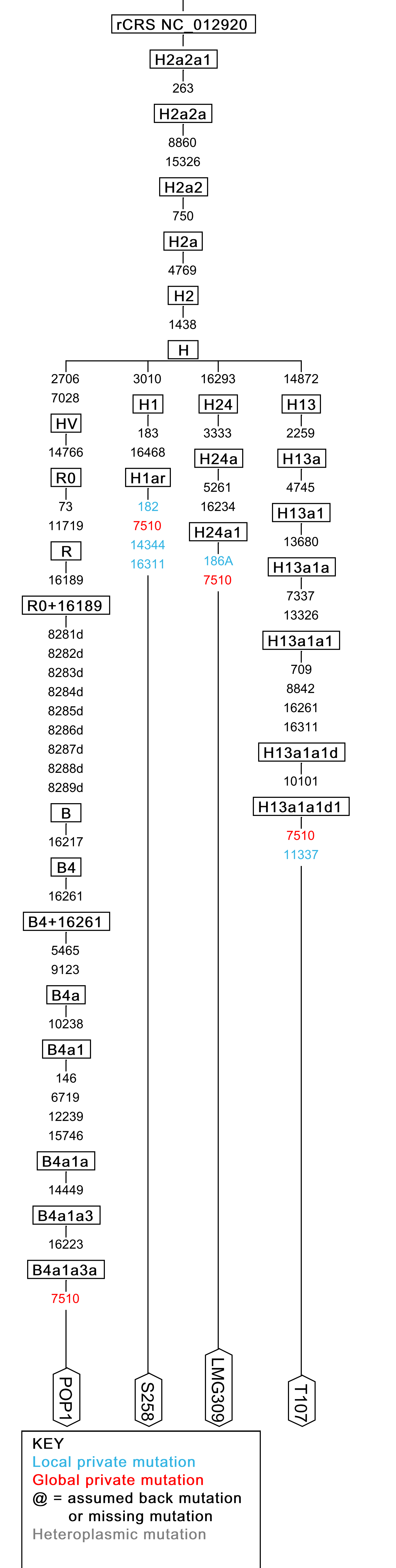
rCRS NC_012920
H2a2a1
263
H2a2a
8860
15326
H2a2
750
H2a
4769
H2
1438
H
2706
7028
HV
14766
R0
73
11719
R
16189
R0+16189
8281d
8282d
8283d
8284d
8285d
8286d
8287d
8288d
8289d
B
16217
B4
16261
B4+16261
5465
9123
B4a
10238
B4a1
146
6719
12239
15746
B4a1a
14449
B4a1a3
16223
B4a1a3a
7510
POP1
3010
H1
183
16468
H1ar
182
7510
14344
16311
S258
16293
H24
3333
H24a
5261
16234
H24a1
186A
7510
LMG309
14872
H13
2259
H13a
4745
H13a1
13680
H13a1a
7337
13326
H13a1a1
709
8842
16261
16311
H13a1a1d
10101
H13a1a1d1
7510
11337
T107
KEY
Local private mutation
Global private mutation
@ = assumed back mutation
or missing mutation
Heteroplasmic mutation
